# Traditional management of ancient Pu'er teagardens in Jingmai Mountains in Yunnan of China, a designated Globally Important Agricultural Heritage Systems site

**DOI:** 10.1186/s13002-023-00598-0

**Published:** 2023-07-01

**Authors:** Wanlin Li, Qing Zhang, Yanxiao Fan, Zhuo Cheng, Xiaoping Lu, Binsheng Luo, Chunlin Long

**Affiliations:** 1 Key Laboratory of Ecology and Environment in Minority Areas, (Minzu University of China), National Ethnic Affairs Commission of China, Beijing, 100081 China; 2 College of Life and Environmental Sciences, Minzu University of China, Beijing, 100081 China; 3 Lushan Botanical Garden, Jiangxi Province and Chinese Academy of Sciences, Lushan, 332900 Jiangxi China; 4 Institute of National Security Studies, Minzu University of China, Beijing, 100081 China

**Keywords:** Ancient teagarden, Biodiversity, Globally Important Agricultural Heritage Systems, *Camellia sinensis* var. *assamica*, Traditional knowledge

## Abstract

**Background:**

Pu'er Traditional Tea Agroecosystem is one of the projects included in the United Nations' Globally Important Agricultural Heritage Systems (GIAHS) since 2012. Against the background of having rich biodiversity and a long history of tea culture, the ancient tea trees in Pu'er have experienced from wild–transition–cultivation for thousands of years, and the local people's knowledge about the management of ancient teagardens has not been rigorously recorded. For this reason, it is important to study and record the traditional management knowledge of Pu'er ancient teagardens and the influence on the formation of tea trees and communities. This study focuses on the traditional management knowledge of ancient teagardens in Jingmai Mountains, Pu'er, and monoculture teagardens (monoculture and intensively managed planting base for tea cultivation) were used as the control, through the community structure, composition and biodiversity of ancient teagardens to respond to the influence of traditional management, and this work with a view to providing a reference for further research on the stability and sustainable development of tea agroecosystem.

**Methods:**

From 2021 to 2022, information on traditional management of ancient teagardens was obtained through semi-structured interviews with 93 local people in the Jingmai Mountains area of Pu'er. Informed consent was obtained from each participant before conducting the interview process. The communities, tea trees and biodiversity of Jingmai Mountains ancient teagardens (JMATGs) and monoculture teagardens (MTGs) were examined through field surveys, measurements and biodiversity survey methods. The Shannon-Weiner (H), Pielou (E) and Margalef (M) indices were calculated for the biodiversity of the teagardens within the unit sample, using monoculture teagardens as a control.

**Results:**

The tea tree morphology, community structure and composition of Pu'er ancient teagardens are significantly different from those of monoculture teagardens, and the biodiversity is significantly higher than that of monoculture teagardens. The local people mainly manage the ancient tea trees mainly using several methods, including weeding (96.8%), pruning (48.4%) and pest control (33.3%). The pest control mainly relies on the removal of diseased branches. JMATGs annual gross output is approximately 6.5 times that of MTGs. The traditional management of ancient teagardens is through setting up forest isolation zones as protected areas, planting tea trees in the understory on the sunny side, keeping tea trees 1.5–7 m apart, as well as consciously protecting forest animals such as spiders, birds and bees, and reasonably rearing livestock in the teagardens.

**Conclusions:**

This study shows that local people have rich traditional knowledge and experience in the management of ancient teagardens in Pu'er, and that this traditional management knowledge has impacted the growth of ancient tea trees, enriched the structure and composition of tea plantation communities and actively protected the biodiversity within ancient teagardens.

## Background

Chinese agriculture has a rich history of thousands of years, with a variety of farming systems and a specialized traditional knowledge [1]. One crop that has gained immense popularity worldwide is tea (*Camellia sinensis* L.). Its distinct flavor, nutrients and reputed health benefits have made it a widely consumed beverage. Tea has been cultivated in China for over 5000 years, solidifying its place as an integral part of the country's agricultural heritage [2, 3]. Tea is not only a popular beverage but also has significant cultural and social importance in China and many other societies. It has been consumed for centuries and is often associated with hospitality, relaxation and health benefits. As a result, the demand for tea is increasing globally, and the tea industry has become a vital source of income for many communities. For developing countries, especially China, the significant expansion of tea cultivation has contributed to the country's economic success in recent years [4–6].

In an effort to increase production and meet the growing demand for tea, monoculture tea plantations have been expanding, but they pose a crisis to the biodiversity and ecosystem services provided by natural forests. Forests are one of the most important ecosystems on Earth, providing not only a large amount of wood, food and medicine, but also carrying many important ecosystem services such as hydrological regulation, soil conservation, climate regulation and landscape beautification [7]. Tea plantations under intensive management are more susceptible to pests and diseases, which may lead to increased use of pesticides and ultimately harm the environment and human health [8]. Therefore, it is important to consider sustainable tea production methods that balance economic interests with environmental and social concerns. This can be achieved through practices such as agroforestry, which involves growing tea alongside other crops and trees, and using natural pest control methods [9, 10]. By adopting sustainable practices, growers can ensure the long-term viability of the tea industry while also protecting the environment and supporting local communities’ economic development.

Yunnan Province in southwestern China is considered to be the botanical origin of tea trees worldwide [11]. It is located at the starting point of the ancient tea horse route and is an important node for the spread of tea culture [5]. The province has the largest area of wild tea tree communities, and the early tea plantations are populated with many ancient tea trees [12]. In addition, three important tea tree types are found in Zhenyuan and Lancang counties in Pu'er City, Yunnan Province, including wild ancient tea trees, which have existed for 2700 years; transitional-type ancient tea tree, which has been in existence for 1700 years; and cultivated-type ancient tea trees, which have existed for 100–1000 years, and the largest area of cultivated-type ancient tea trees remains in Jingmai Mountains of Pu'er, with 18 km^2^ [13, 14]. The term "wild-type tea tree" refers to a tea tree that has been preserved by natural selection in a specific area for an extended period; as tea trees continued to evolve, this type of tea tree gave rise to "transitional-type tea tree" that exhibit both wild and cultivated characteristics; the "cultivated-type tea tree" is a new type of tea tree that has been created by humans through the process of selection, cultivation and domestication of the wild-type tea tree. Therefore, with the rich tea germplasm resources and the long history of tea culture background the Pu'er Traditional Tea Agroecosystem is a Globally Important Agricultural Heritage System (GIAHS) that was recognized by the Food and Agriculture Organization (FAO) of the United Nations in August 2012 [15]. The GIAHS program was launched by the FAO in 2002 with the aim of identifying agricultural systems that are of global importance. The program seeks to preserve landscapes, agrobiodiversity and traditional knowledge, while also promoting sustainable development through the application of dynamic conservation principles [16].

The Pu'er Traditional Tea Agroecosystem is the largest tea forest plantations in the world, established by village ancestors thousands of years ago. Ancient teagardens, aggregated or scattered in the natural forest, have *Camellia sinensis* var. *assamica* over 100 years old [17]. Ancient teagardens can contain three types of ancient tea trees: wild, transitional, or cultivated. The cultivated-type ancient tea trees are used by people and the largest by area are on Jingmai Mountains. Early domestication and cultivation of the ancient tea trees were conducted by the Bulang and Dai peoples, and after extended practice and management over generations, an extraordinary cultivated ancient teagarden landscape developed. Therefore, these Jingmai Mountains ancient teagardens (JMATGs) are one of the important heritage sites of Pu'er Tea Agroecosystem.

The biodiversity of JMATGs has been well studied over the past two decades. In 2005, the biodiversity of JMATGs was found to be close to that of natural forests and higher than that of newer monoculture teagardens [18]. The carbon stock of JMATGs was shown to be significantly higher than that of new monoculture teagardens [19], and most recently researchers determined that the local traditional culture, including village rules and regulations, folklore and festivals, contained an awareness of the conservation of JMATGs [20]. JMATGs have been managed by the Bulang and Dai people for thousands of years. These ancient teagardens are rich in genetic and cultural diversity, but how they have been managed is still unclear. With the rise of digitalization and technology, the dissemination and inheritance of traditional knowledge is facing great challenges and threats. The fact that the Bulang and Dai people have been able to peacefully coexist with the JMATGs for thousands of years and maintain a high level of biodiversity is an indication that their traditional management is able to support the sustainable development of the teagardens. In this study, one of the most representative ancient teagardens in Pu'er, the ancient teagardens in Jingmai Mountains, was used as the research site, and the traditional management methods and related traditional knowledge of ancient teagardens were investigated to prevent the traditional management knowledge of ancient tea plantations accumulated over hundreds of years from disappearing into the world forever at some point in the future.

## Methods

### Study area

Pu'er is an administrative unit located in the southern region of Yunnan Province, consisting of 98.3% mountainous terrain. It is situated between 22°02′N–24°50′N and 99°09′E-102°19′E, with the Tropic of Cancer passing through the middle of the city. Additionally, Pu'er shares borders with Vietnam, Laos and Myanmar [21]. Pu'er has a diverse range of altitudes, spanning from 317 to 3,370 m. The average annual temperature is 19.5 °C, and the yearly rainfall typically is between 1100 and 2780 mm and belongs to the subtropical mountain monsoon climate. The forest coverage is extensive, accounting for approximately 75% of the area, and includes various types of vegetation such as seasonal rainforest, mountain rainforest, monsoon evergreen broad-leaved forest, warm coniferous forest and other forest types [22]. This vast array of flora contains almost one-third of China's species. Pu'er is renowned for its abundant tea resources, and its rich history of tea culture. The region boasts 787.06 km^2^ of wild-type ancient tea trees communities, 126 km^2^ of cultivated-type ancient teagardens and 1291.98 km^2^ of modern teagardens [23].

**Fig. 1:**
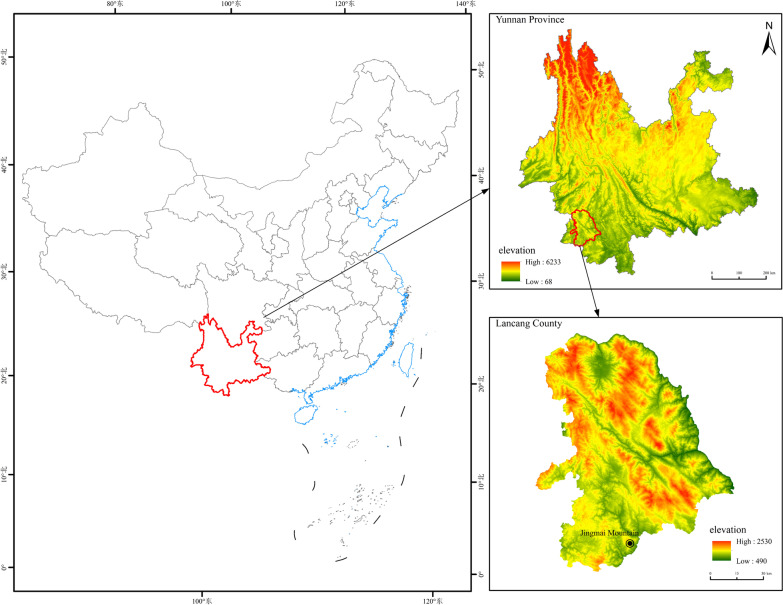
Location of the study area in Yunnan Province, China

**Fig. 2:**
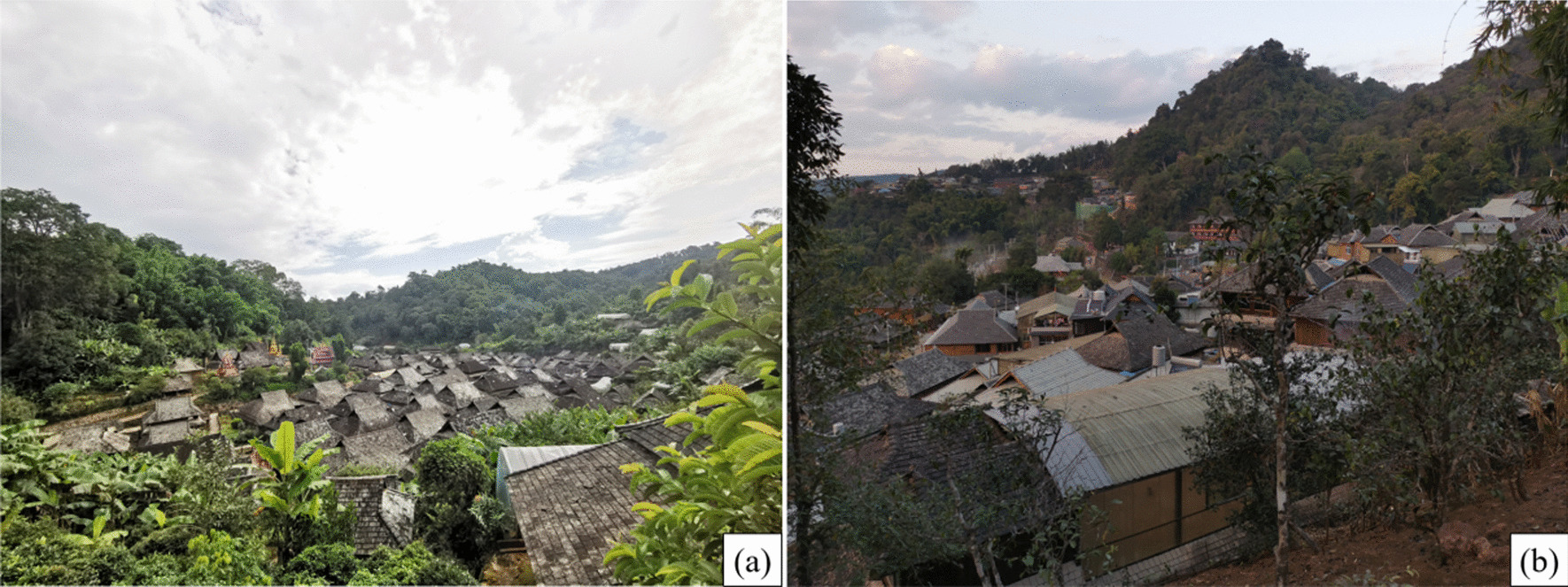
The villages in JMATGs. **a** One of the Dai Village, named Nuogang; **b** One of the Bulang Village, named Mangjing Shangzhai; (**a**, **b**) Photos by Wanlin Li

The JMATGs are the largest among all of the distribution regions with ancient teagardens in Yunnan Province. This area covers more than 6.67 km^2^ (Fig. 1). Jingmai Mountains belongs to the end of the Nushan Mountains range, and numerous small mountain ranges formed in between, with a total area of about 100 km^2^. Jingmai Mountains are located in the southwest border of China's Yunnan Province, adjacent to Menghai County in Xishuangbanna to the east and Myanmar to the west, and is the border between Xishuangbanna, Pu'er and Myanmar [24]. It is located in Lancang Lahu Autonomous County, Pu'er City, between 99°59′14″N–100°03′55″N and 22°08′14″E–22°13′32″E. The average altitude of 1500 m, an average annual rainfall of 1800 mm and an average annual temperature of 18 ℃ [25]. There are 14 traditional villages in the Jingmai Mountains, which belong to two administrative villages, Jingmai Village and Mangjing Village, where the Bulang, Dai, Lahu and Hani ethnic groups live, and the Bulang and Dai are the majority in this region. The people living in the Jingmai Mountains mainly rely on tea production as their main economic source. The Jingmai Mountains is well-suited to grow *Camellia sinensis* var. *assamica*, with red soil and a subtropical environment that supports many evergreen broad-leaved plants (Fig. 2).

### Data collection

From January 2021 to August 2022, four surveys of the JMATGs were conducted in 48 days. During these surveys, six villages were visited, including Jingmai Daizhai, Mangjing Shangzhai, Mangjing Xiazhai, Mengben, Nuogang and Wengji. This study employed ethnobotany and ecology methods to explore, document and analyze the traditional management techniques, indigenous knowledge and biodiversity of JMATGs. In order to showcase the biodiversity of JMATGs, they were compared with monoculture teagardens (MTGs). A sampling survey was used to analyze the plant diversity of monoculture teagardens.

In this investigation of biological community composition of JMATGs, MTGs in the same area were selected as controls. To obtain accurate measurements, the height of tea trees was measured using the Haguang direct-reading tree height meter CGQ-1, and the tree circumference and crown width were measured using a Hoffmann 0.5 mm precision tape measure. Additionally, the age and species of the tea tree are based on the label on the tea tree, as determined by the Tea Research Institute. To investigate the species and number of plants in the ancient teagardens, vegetation sampling plots investigations were used. JMATGs and MTGs were each set up with five sample squares, and statistical analysis was conducted based on a 400 m^2^ (20 × 20 m) area for trees, shrubs, parasitic and epiphytic plants, and a 1 m^2^ (1 × 1 m) area for herbs. To compare the morphology of tea plant in JMATGs and MTGs, three tea plant were randomly selected from each of the sample squares JMATGs and MTGs to measure the spacing, height and crown diameter of the tea plant.

**Fig. 3:**
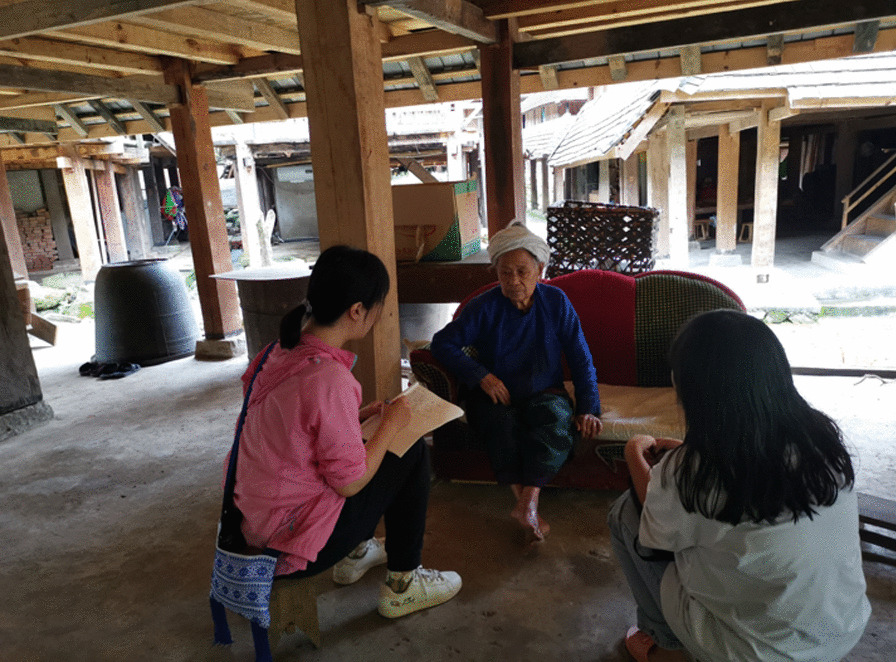
Ethnoecological surveys of traditional management in JMATGs

The semi-structured interview method was used to interview JMATGs conventional management and traditional management knowledge (Fig. 3). All field studies were carried out with prior informed consent of all persons interviewed. The questions used for semi-structured interviews are: 1. How to manage your ancient teagardens? 2. How often to manage the ancient teagardens? 3. When to take care of your ancient teagardens? 4. How well the ancient teagardens are managed? 5. How to manage diseases in ancient teagardens? 6. How frequently are chemical fertilizers and pesticides used in ancient teagardens? 7. What is the traditional knowledge about the management related to ancient teagardens? The surveys involved 93 participants, consisting of 53 men and 40 women (Table 1). The majority of those responsible for managing the JMATGs were middle-aged and young individuals. Specifically, the demographics of the subjects were 67.7% and within the age range of 20–50. 58.1% had received primary school education. In terms of ethnicity, the Bulang and Dai groups were the most represented, accounting for 51.6% and 32.3% of the interviewees, respectively.

**Table 1:** Demographic characteristics of the interviewees

Characteristics	Number of respondents	%
Sex		
Male	53	57
Female	40	43
Age		
20–29	13	14
30–39	34	36.6
40–49	16	17.2
50–59	8	8.6
60–69	12	12.9
>70	10	10.7
Ethnic group		
Bulang	48	51.6
Dai	30	32.3
Hani	2	2.1
Han	9	9.7
Lahu	2	2.1
Yao	1	1.1
Yi	1	1.1
Formal education		
Illiterate	12	12.9
Primary school	54	58.1
Middle school	20	21.5
High school	3	3.2
Junior college	3	3.2
Bachelor degree	1	1.1
Main occupation		
Farmer	90	96.7
Local official	1	1.1
Teacher	1	1.1
Forest ranger	1	1.1

### Data analysis

The biodiversity of plant communities in teagardens was measured with the Shannon-Weiner (H), Pielou (E) and Margalef (M) indices [26]. The Shannon-Weiner index can be used to measure the diversity and abundance of species in an ecosystem. The higher the index value, the higher the species diversity. The Pielou index is a measure of how evenly the number of individuals of a species is distributed in a community. Margalef index reflects community species richness. It refers to the number of species in a community or environment and also indicates the degree of species richness in a biotope. These measure formulas are:

$$\begin{gathered} H = \left( {N\lg N{-}\Sigma n\lg n} \right)/N \hfill \\ E = H/\left( {\ln N} \right) \hfill \\ M = \left( {S{-}1} \right)/\left( {\ln N} \right) \hfill \\ \end{gathered}$$

where *N* is the total number of individual plants in each quadrat, *n* is the number of individual plants in each quadrat and *S* is the number of species of plants in each quadrat. The significance of the community diversity index of different teagardens was analyzed by SPSS statistical software and compared by the LSD method (*P* = 0.05).

## Results

### Community composition and biodiversity indices of JMATGs

**Fig. 4:**
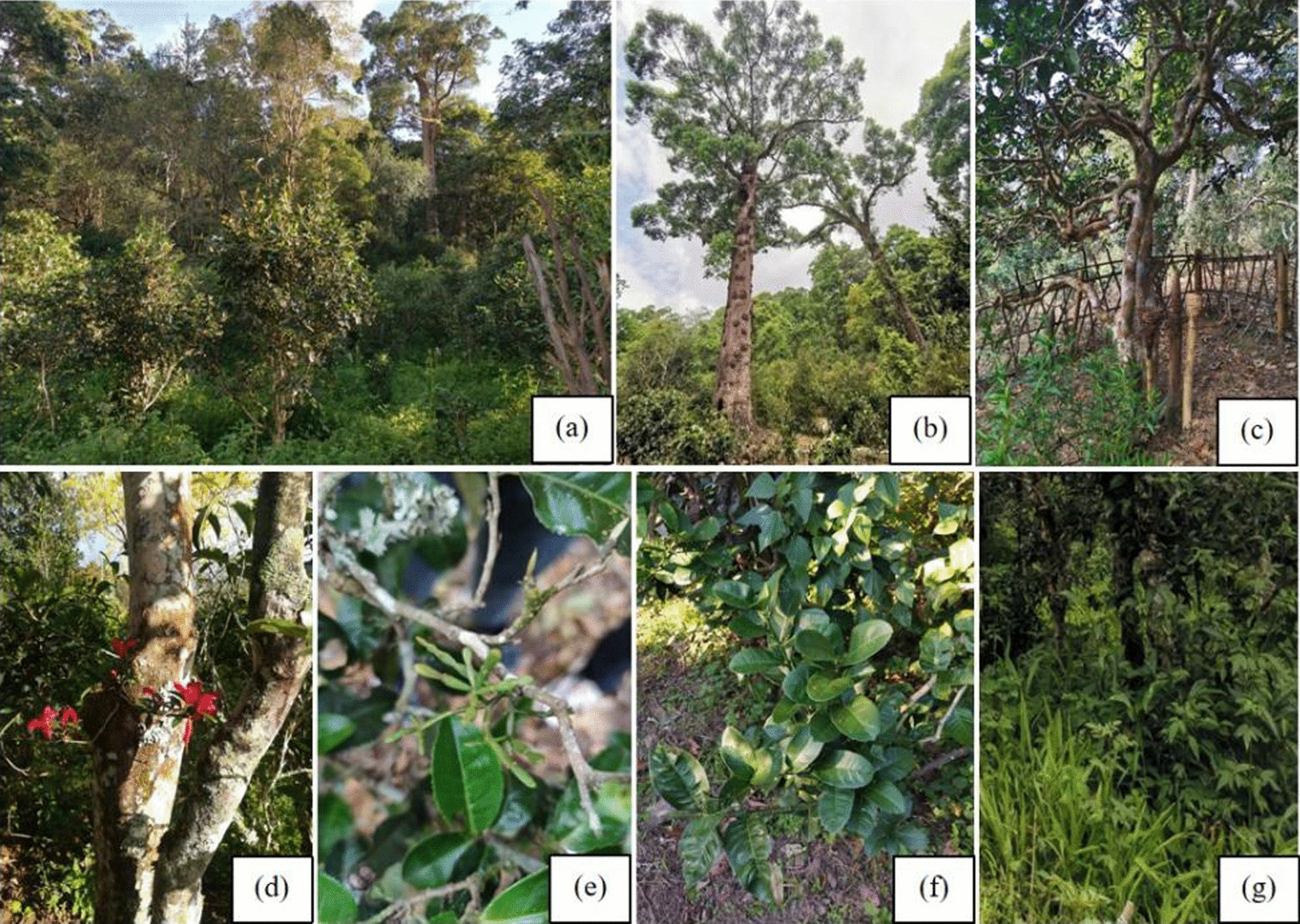
Plants with different community structures in JMATGs. **a** Part of an JMATG; **b** Tall trees in an JMATG; **c** Old tea trees in JMATGs; (**d**, **e**) Parasitic and epiphytic plants on ancient tea trees; **f** Shrubs in JMATGs; **g** Herbs in JMATGs; (**a**–**c**, **e**–**f**) Photos by Wanlin Li; **d** Photo by Yuanyan Zhao

The community composition of JMATGs consists of five vegetation layers, including tall tree, tea tree, shrub, herb and interlayer with epiphytic and parasitic plant (Fig. 4). The upper tall trees mainly consist of Anacardiaceae, Fabaceae and Lauraceae. Tea trees are in the Theaceae family, which occupies the dominant population in the plant community of JMATGs. The shrub layer includes smaller trees and shrubs belonging to Moraceae, Primulaceae, Verbenaceae, among other families. The ground layer consists mostly of plants belonging to Apiaceae, Lamiaceae, Poaceae and Rubiaceae. Interlayer plants mostly belong to Loranthaceae, Gesneriaceae and Orchidaceae.

**Fig. 5:**
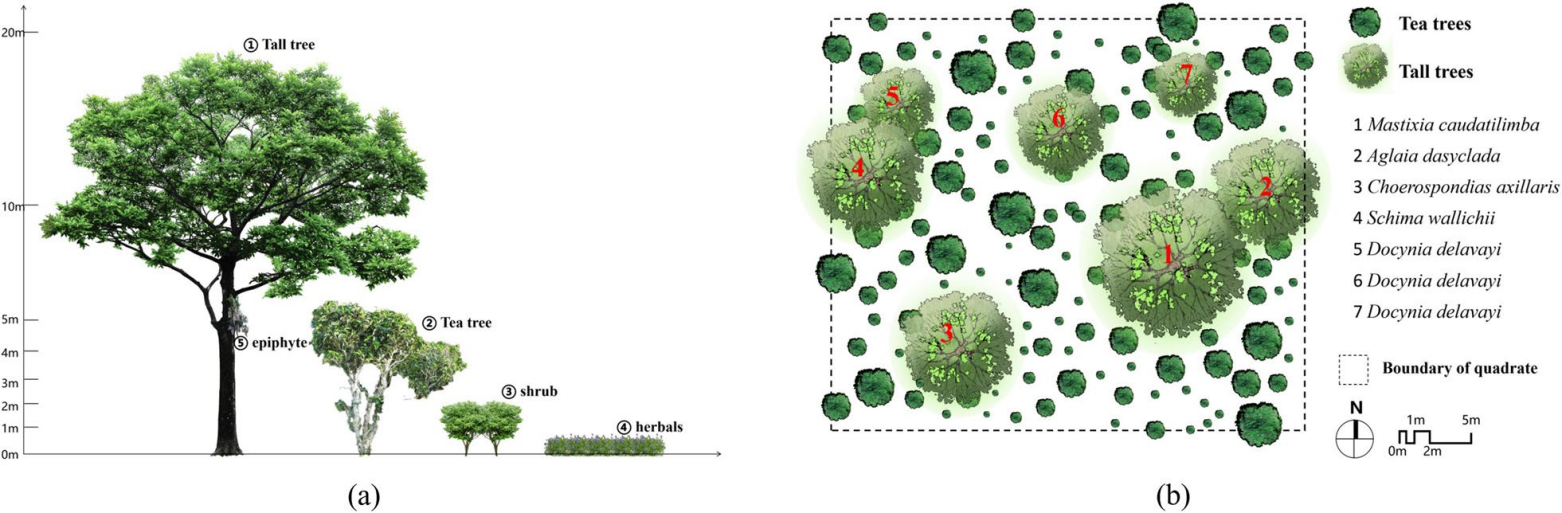
Community structure of JMATGs. **a** The side anatomical diagram of JMATG; **b** The overhead view of JMATG

To study the community structure of the ancient teagardens more directly and succinctly, the side vertical anatomy diagram and the front overhead view were drawn (Fig. 5). The height range of tall trees in the top layer is mainly 10–25 m, the tea trees layer 2–8.5 m, the shrub layer 1.5–3 m and the herb layer 0–1.5 m. Parasitic and epiphytic plants usually grow on the trunks or branches of trees. In the aerial view of an ancient teagarden quadrate, the canopy of tall old tea trees can easily be observed due to their large canopy coverage. However, the distribution of tea trees scattered under the tall trees is incomplete.

The Shannon–Wiener (H), Pielou (E) and Margalef (M) indices of JMATGs were significantly different from the MTGs (controls) by measuring the biodiversity indices (Table 2). S in the table refers to the total number of species in the JMATGs and MTGs within the five samples, and the number of species in the JMATGs was five times higher than that in the MTGs. N is the average number of individuals in the five sample squares, and the number of individuals per unit sample square in MTGs is more than four times that in JMATGs.

**Table 2:** Biodiversity indices (mean ± SE) of plant communities in MTGs and JMATGs

Type of teagarden	S	N	Shannon–Wiener index (*H*)	Pielou index (*E*)	Margalef index (*M*)
MTGs	29	1791	1.43 ± 0.32^b^	0.5 ± 0.11^b^	2.27 ± 0.33^b^
JMATGs	136	400	3.56 ± 0.14^a^	0.60 ± 0.03^a^	10.55 ± 0.76^a^

^a, b^ Different letters in the same row indicate difference at 0.05 level

### Difference between JMATGs and MTGs

**Fig. 6:**
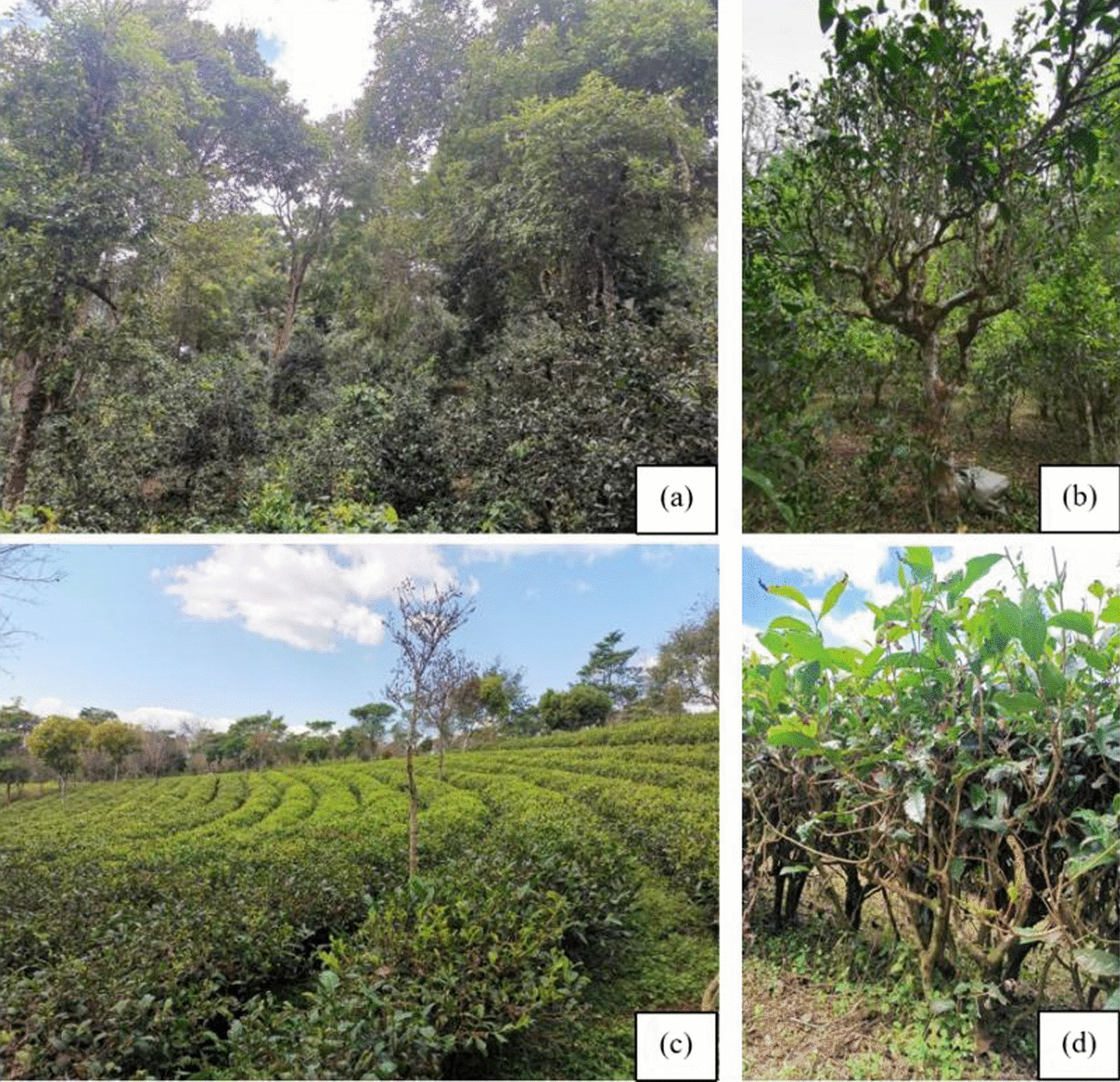
The different teagardens landscape and tea plants. **a**, **b** JMATGs and ancient tea trees; **c**, **d** MTGs and tea trees or shrubs. **a**–**d** photos by Wanlin Li

There are obvious differences between JMATGs and MTGs in terms of landscape, community structure, tea tree planting and morphology. In the JMATGs, the tea species is *Camellia sinensis* var. *assamica*. Morphologically *Camellia sinensis* var. *assamica* are trees with distinct trunks, most of which are 2.5–8.3 m in height and 1.2–4.5 m in diameter of tea tree canopy (Table 3). Most of the tea trees growing in the JMATGs are ancient ranging from 100 to 1000 years old. There are also some small tea trees in the gardens. For tea tree planting, farmers choose to plant under shade trees and the distance between tea saplings is 1.5–7 m. The overall landscape of the ancient tea plantation resembles a natural forest (Fig. 6a).

**Table 3:** The difference between MTGs and JMATGs

Characteristic	MTGs	JMATGs
Landscape	Monoculture shrubs	Forest
Community composition	Monoculture tea planting	Arbor, shrub, herb, parasitic and epiphyte coexisting
Agricultural type	Intensive agricultural	Mixed agroforestry
Habit	Shrubs	Trees
Age	10–50 years	100–1000 years
Tea plant spacing/m	0.1–0.3^*^ / 0.19 ± 0.07^b**^	1.5–7^*^ / 4.38 ± 1.59^a**^
Species	*Camellia sinensis* var. *sinensis* or *C. sinensis* var. *assamica*	*Camellia sinensis* var. *assamica*
Tea plant height/m	0.62–0.95^*^ / 0.81 ± 0.11^b**^	2.5–8.3^*^ / 5.06 ± 1.87^a**^
Diameter of tea plant canopy /m	0.45–0.7^*^ / 0.57 ± 0.1^b**^	1.2–4.5^*^ / 2.9 ± 0.85^a**^
Tea plant trunk	No	Yes

^a, b^ Different letters in the same row indicate difference at 0.05 level

^*^Refers to the minimum to maximum value range

^**^Refers to the mean ± SE

In the MTGs, the trees are shrubby, dense and planted neatly in rows, forming a recognizable landscapes (Fig. 6c). Tea saplings can be planted on hillsides, forming stepped teagardens, and the species is either *Camellia* *sinensis* var. *sinensis* or *Camellia* *sinensis* var. *assamica*. These younger tea trees have a shrubby habit with no obvious main trunk, with a height of only about 0.62–0.95 m (Fig. 6d). Comparing the tea plant space, height and canopy diameter of JMATGs and MTGs, significant differences were found between the ranges.

### Traditional management of ancient tea trees

**Fig. 7:**
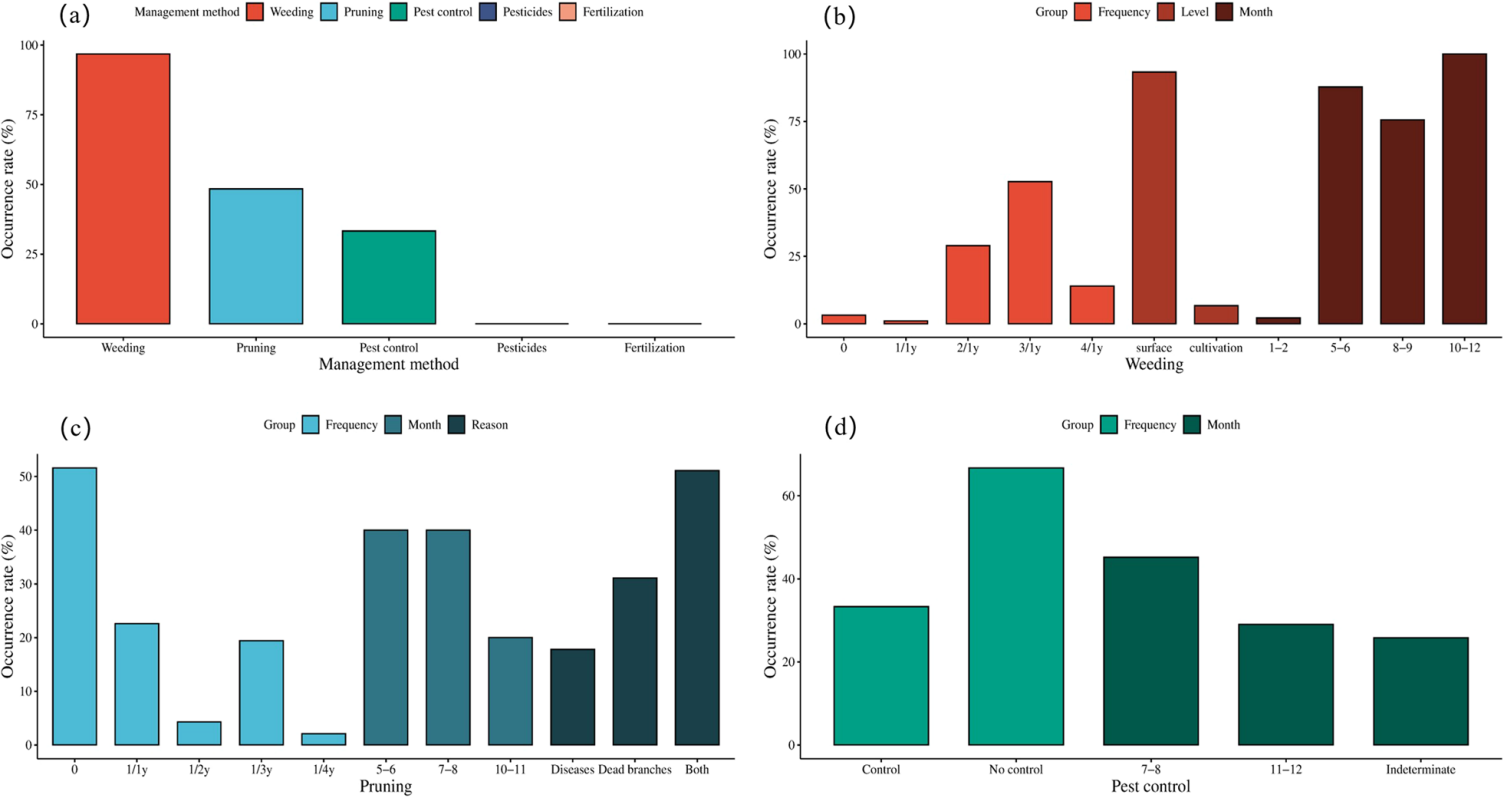
Management of JMATGs. **a** Management methods in JMATGs; **b** Frequency, level and time of weeding in JMATGs; **c** Frequency, time and reason of pruning management in JMATGs; **d** Frequency of pest control and time of tea tree diseases occurrence in JMATGs

The management of JMATGs mainly focuses on three jobs: weeding, pruning and pest control (Fig. 7a). According to the informants, weeding was performed by 96.8% of tea farmers, pruning by 48.4% and pest control by 33.3%. Fertilizers and pesticides are not used in the management of JMATGs. In weed management, only 3.2% of tea farmers did not weed; 1.1% weeded once a year; 29% twice a year; 52.7% three times a year; and 14% four times a year (Fig. 7b). The frequency of weeding three times a year is the largest number. The selection of weeding time was also different. Only 2.2% of farmers weeded from January to February; 87.8% from May to June; 75.6% from August to September; and 100% from October–December. Therefore, most people choose May–June, August–September and October–December for the weeding. In the degree of weeding, 93.3% of people weeded on the soil surface, while the others chose to turn the soil.

In pruning management, 51.6% of farmers prefer not to prune (Fig. 7c). Among those who pruned, 22.6% pruned once a year; 4.3% once every two years; 19.4% once every three years; and 2.1% once every four years. Pruning in May to June was performed by 40% of the respondents, July to August 40%, and October to November 20%. There were three main reasons farmers prune tea trees (Fig. 6): 17.8% of informants removed only diseased branches; 31.1% removed only dead branches; and 51.1% removed both diseased and dead branches. One-third of the information reporters chose to control tea tree pests and diseases, with control methods relying mainly on the removal of diseased branches. The informants mentioned that tea tree disease in JMATGs mainly occurred in either July–August (45.2%), November–December (29%), or the time was not fixed (25.8%) (Fig. 7d).

### The economic output of JMATGs and MTGs

**Fig. 8:**
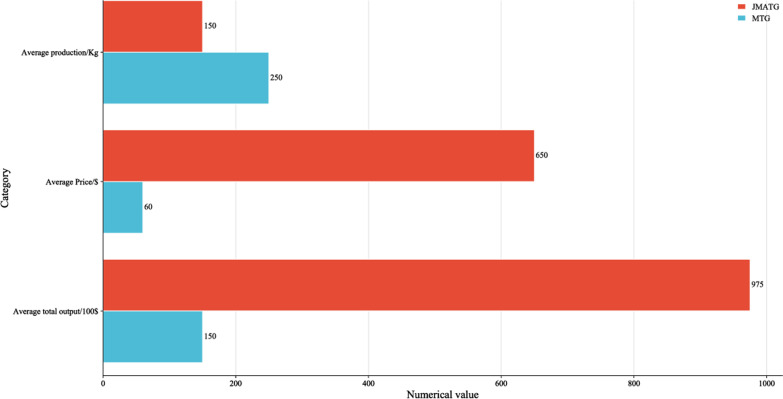
Annual production and output value of JMATGs and MTGs

Economic production value of JMATGs and MTGs was learned through survey interviews. Because tea production and prices do not vary much from year to year, we consulted the production volume (Kg) and unit price ($/Kg) per 666.67 m^2^ of JMATG and MTG in 2022 and calculated the total value. JMATG's dry tea production per 666.7 m^2^ in 1 year is 100–200 kg, with an average value of 150 kg. MTG is 200-300 kg, with an average value of 250 kg. Therefore, the tea production of MTG is more than 1 times that of JMATG. However, the unit price of JMATG's dry tea ranged from 300–1000 RMB, with an average price of 650 RMB. MTG ranged from 40–80 RMB, with an average price of 60 RMB. It can be calculated that the annual average gross output value per 666.67 m^2^ of JMATG is about $97,500 and MTG is $15,000. 6.5 times of JMATG than MTG (Fig. 8).

### Traditional management of JMATGs

**Fig. 9:**
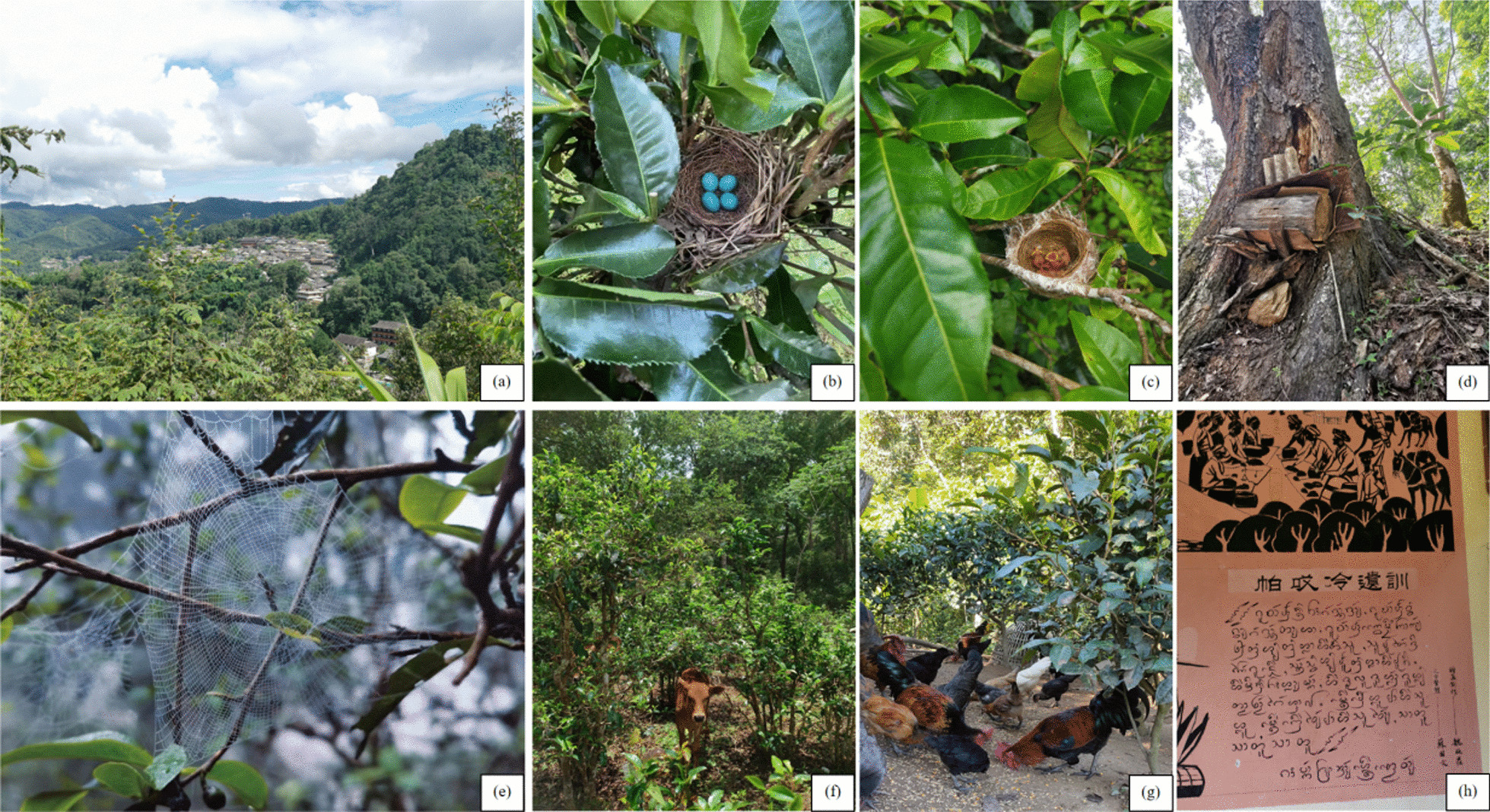
Traditional management of JMATGs. **a** The village and forest in the JMATGs;** b**,** c** Nests, eggs and nestlings on a tea tree; **d** A beehive in a JMATG; **e** A spider web on a tea tree; **f** Villagers herded cattle in an JMATG; **g** Chickens in an JMATG; (**a**, **b**, **c**, **d**, **h**) photos by Wanlin Li; (**e**, **f**) photos by resident Wanxian Yue; **h** photo by Kong Ye

Local residents manage JMATGs according to topography, climate, species conservation and the growth characteristics of tea trees. Regarding topography, the tea plantation should not be too dense, and a primeval forest of about 40 m in width is reserved as a buffer zone between teagardens and villages (Fig. 9a). Considering the climate, *Camellia* *sinensis* var. *assamica* is grows well between 1400 and 1600 m above sea level, and this improves the tea quality. At higher altitude, tea plants have improved growth and quality. Finally, depending on the amount of sunlight, tea trees are typically planted in the direct sun.

*Camellia sinensis* var. *assamica* tea trees are typically grown under large trees, creating partial shade and increasing the flavor of the tea. The tea trees are allowed to grow naturally with minimal disturbance except for pruning side branches. To protect plant species, the local Bulang and Dai people respect nature through their Buddhist beliefs and village rules and regulations, thereby protecting natural plants, animals and local ecosystems (Fig. 9h). In the traditional cultural management of teagardens, plants are consciously preserved and protected because of their edibility, medicinal functions and landscape functions. Animals such as spiders, bees and birds in teagardens are also consciously protected because they are beneficial to the plants, such as pollinators.

The most common animal kingdom species found on ancient tea trees are spiders, like *Euophrys frontalis, Plexippus paykulli* and *Telamonia dimidiata*. Spider webs are consciously kept and protected in tea plantations because of their role in catching pests (Fig. 9e). Bees cross pollinate plants, increase fruitfulness and produce honey that provides more nutrition for local people. The farmers typically place wooden barrel beehives on large trees or on hillsides (Fig. 9d). The role of birds as seed dispersers and pest reduction is valued by local people (Fig. 9b,c). The locals sometimes raise animals, such as chickens and cattle, in the teagardens (Fig. 9f,g). Chickens or other poultry are located in tea plantation that are closer to their coops, while cattle are found in teagardens farther away from their barn.

## Discussion

### Effects of traditional management on tea trees and communities of ancient teagardens

JMATGs refers to the area of many tea trees over 100 years old are planted on the Jingmai Mountains and are traditionally managed by local people. MTGs refers to the tea growing area where monoculture tea trees are under 100 years old planted and managed intensively. Kfoury and colleagues’ study of tea quality at high altitude (1400 m) and low altitude (600 m) showed that teas produced at high altitude were more sweet, floral and honey-like, while teas produced at low altitude were more bitter. JMATGs average altitude of 1500 m provides evidence of good tea taste [27]. Shade trees also play an important role in improving the quality of tea, improving the nutritional and sensory quality of tea leaves [28, 29]. In tea plantation management studies, it was found that high synthesis of glucose and high accumulation of catechins and their derivatives in unpruned tea trees exhibited strong photosynthetic activity and that their unpruned teas had lower bitterness and astringency [30, 31].

The community of JMATGs can be divided into five vertical levels that have a close relationship with traditional management. As the ancient tea trees have been planted under larger trees in the natural forest, the distance between tea trees is in the range of 1.5 to 7 m (Table 3), preserving enough space for the surface layer plants to grow. The retention of large trees provides parasitic and epiphytic plants an ideal environment to grow. This not only enriches the composition of the ancient teagardens community, but also provides more space for plants, animals and microorganisms [32]. The trees in the ancient teagardens provide water to the local communities and regulate the climate; the different plants provide food, timber and medicine and make an inescapable contribution to the local architecture and provide energy and nutrition [33]. Meanwhile, traditional management provides a more protective role for such tea forest ecosystems and more sustainable information for other agroforestry or agroecosystems [34].

### Effects of traditional management on biodiversity of ancient teagardens

Traditional management of Pu'er ancient teagardens has played a significant role in maintaining their ecosystem services while also offering more possibilities for biodiversity conservation [35]. The number of species in the survey sample of ancient tea plantations under traditional management is more than four times higher than that of MTGs, and the biodiversity index also shows that the biodiversity of JMATGs is significantly higher than that of MTGs (Table 2). In particular, traditional management can play an important role in growing and maintaining crop varieties and livestock breeds, protecting and using edible and medicinal plants and preserving extraordinary landscapes [36, 37]. About 25% of the world’s land is settled on by local communities, many who have played a critical role in preserving much of the world's biodiversity for thousands of years [38]. Over the past few decades, dramatic changes in land use have occurred, such as the shift from complex natural ecosystems to those with simplified management, and intensification of resource use, including the use of more agrochemicals [39]. These changes have resulted in biodiversity loss occurring on an unprecedented scale worldwide, and agricultural intensification is the main driver of this global change [40].

When global climate change hinders biodiversity conservation, local communities using traditional management lose biodiversity at a slower rate than other areas [37, 41, 42]. Resilience of conventionally managed agricultural systems to climate hazards is closely related to high levels of on-farm biodiversity, based on observations of agricultural performance after 20 years of extreme climate events [43]. Agricultural intensive management has transformed some forests, including or primary forests, into monoculture tea plantations, thus decreasing biodiversity [44]. Monoculture agriculture not only reduces biodiversity, but can also be used to spread diseases due to monoculture planting, which may result in large areas and high levels of disease occurrence, which is not conducive to disease control efforts [45]. In Pu'er ancient teagardens, the tea tree diseases occur from time to time, but only 33.3% of the information reporters chose pest control (Fig. 6). The reason one is the disease situation is not serious or not a widespread infection, and the other hand is the impact on tea tree growth is not great, so most people choose not to treat the disease [46]. Therefore, the rich biodiversity of the ancient tea plantation may play an important role in regulating the ecosystem, including resisting the occurrence of large scale outbreaks of pests and diseases [47–49].

### Impact and suggestion of traditional management of ancient teagardens for the economic development of local communities

The economic value of JMATGs tea is 6.5 times higher than MTGs (Fig. 8), this value is an approximate estimate, the actual situation may be more than 6.5 times. First, JMATGs have no fertilizer or pesticide inputs, and labor costs are relatively low than MTGs. Most MTGs are not low cost in fertilizer and pesticide inputs, and the annual use of pruning and herbicides in tea trees also increases the cost of inputs, and we only counted the outputs, not the input costs. Therefore, the economic value of tea in JMATGs is not just 6.5 times higher than MTGs. Second, the tea picking in JMATGs occurs 2 times a year (spring and autumn), while MTGs tea picking occurs 3 times a year (spring, summer and autumn), of which summer is the season with the most rainfall, and therefore, the yield is also greater than spring and autumn, which is why the annual yield of MTGs is greater than JMATGs.

The outstanding landscape of Pu'er ancient teagardens received the attention of domestic and foreign experts and tourists, the quality of tea leaves in the ancient teagarden was also highly praised by tea lovers. Therefore the economic value of tea has also risen. And under the influence of GIAHS, tourism has also started to develop in Jingmai Mountains [50]. With the increase of hotels and restaurants, the sale of local products (like tea, honey, dendrobium, chili powder and wild fungus) and ethnic costumes has added a series of economic income to the local community in Jingmai Mountains [51].

As the economy improves and grows there are also many implications. First, the increase of human traffic will certainly increase the consumption of resources and waste products, how to reasonably use and dispose of them in order not to damage the local ecological environment is something that needs to be carefully considered [52]. Secondly, in order to improve the accommodation experience and the convenience of transportation, building houses and roads should be implemented without affecting the local environment, and it is recommended to have a professional assessment before proceeding [53]. Thirdly, because Jingmai Mountains is far from the town, you must drive yourself up the mountains. Tourism, more and more people buying tea, also means more and more cars, and it is not clear whether the exhaust from cars has an impact on the tea forest ecosystem [54]. Finally, in order to increase economic income, the focus is put on the collection, production and sale of tea, while neglecting the learning and transmission of local traditional culture, which should attract attention and concern.

### Crisis and challenges of traditional management of JMATGs

As the tea economy grows, more and more people are focusing on the yield and economic value of tea production [55]. To increase tea production to meet the commercial demand for tea, the modernized and intensive monoculture teagarden business model prevails [56]. This also happened in the former JMATGs. Due to the low value of tea and the lack of self-confidence in traditional management, the local people have converted few ancient teagardens and forests into monoculture teagardens under the influence of intensive agricultural operations and the popular trend of monoculture tea plantations. It did not improve the quality of tea, but on the contrary, it had a negative impact on the traditional tea agroecosystem [57]. With timely government and expert visits and prevention, and in 2012, the Pu'er Traditional Tea Agroecosystem was recognized as a GIAHS project, the JMATGs has received much attention from experts and scholars nationally and internationally as one of the most important heritage sites of the system [11]. The economic value of tea in ancient teagardens has also increased, thus also increasing the cultural confidence of traditional management. To prevent the local residents from destroying the biodiversity and ecological environment in the ancient teagardens due to the improvement of the tea economy, a series of protection measures have been formulated, such as the "Regulations on the Protection of Jingmai Mountains in Lancang Lahu Autonomous County, Yunnan Province" and the "Regulations on the Protection of Ancient Tea Tree Resources in Pu'er City" [58, 59]. However, it is unclear whether these protection measures are entirely beneficial to the conservation of the ancient teagardens in Jingmai Mountains, and whether they will form an over protection.

JMATGs is not only an important heritage site of Pu'er Traditional Tea Agroecosystem of GIAHS, but also in 2023 is being declared as a World Heritage Site [60]. The Dai, Bulang, Hani, Lahu people living in the Jingmai Mountains have created extraordinary ancient teagardens landscapes through the experience and traditional knowledge of tea plantation and teagardens management accumulated over hundreds of years. These centuries of peaceful coexistence with the ancient teagardens include not only the traditional knowledge related to tea trees and teagardens, but also many other traditional cultures and knowledge. These traditional cultures and knowledge are now facing the replacement of new knowledge and skills and disappearing with the departure of the older generation. This management-related traditional knowledge is passed down through generations by word of mouth and hands-on teaching of the practical processes, and there is still much traditional knowledge that remains untapped, recorded and protected. These traditional knowledge play a non-negligible role in the biodiversity conservation, ecosystem stability and sustainable development of ancient teagardens [61–63].

## Conclusion

This study recorded and summarized the traditional management of JMATGs, which included ancient tea trees and ancient teagardens. The traditional management of ancient tea trees mainly focuses on weeding practiced by 96.8% of tea farmers, followed by pruning, practiced by 48.4%. The main purpose of pruning is to remove the dead and diseased branches. Under the traditional management of ancient tea trees, the tree height is between 2.5 and 8.3 m and the canopy width can reach 1.2–4.5 m (Table 3). The understory planting of tea trees makes the community structure of ancient teagardens rich, and it can be divided into five levels: tall tree, tea tree, shrub, herb and parasitic and epiphytic plant layers. Isolation reserves and sufficient space between tea trees provides a habitat for more organisms and promotes the conservation of biodiversity and ecosystem stability. JMATGs are one of the important heritage sites of Pu'er Traditional Tea Agroecosystem listed in the GIAHS. This has brought great benefits to the economic value of JMATGs tea, further affirming the local management of traditional teagardens. Its traditional management is the experience of the local Bulang and Dai people who have been practicing it for thousands of years. This study provides a record of this important traditional management knowledge and reference material for future biodiversity conservation and sustainable development of ancient teagardens.

## Data Availability

All data, materials and information are collected from the study sites.
